# Elevated pressure downregulates ZO-1 expression and disrupts cytoskeleton and focal adhesion in human trabecular meshwork cells

**Published:** 2011-11-16

**Authors:** Xuejiao Yang, Bingqian Liu, Yujing Bai, Min Chen, Yiqing Li, Mengfei Chen, Yantao Wei, Jian Ge, Yehong Zhuo

**Affiliations:** 1 State Key Laboratory of Ophthalmology, Zhongshan Ophthalmic Center, Sun Yat-sen University, Guangzhou, China; 2 Key Laboratory of Vision Loss and Restoration, Ministry of Education, Department of Ophthalmology, Peking University People’s Hospital, Beijing, China; 3 Department of Opthalmology, the second affiliated hospital of Zhejiang University, Hangzhou, Zhejiang, China

## Abstract

**Purpose:**

To investigate the effect of elevated hydrostatic pressure on the expression and distribution of zonula occludens-1 (ZO-1), and its effect on cytoskeleton and focal adhesion in immortal human trabecular meshwork cells (iHTM) and glaucomatous human trabecular meshwork cells (GTM_3_).

**Methods:**

iHTM and GTM_3_ were exposed to 60 mmHg hydrostatic pressure for 6, 12, and 24 h. As a control, the cells were incubated simultaneously in a conventional incubator. Morphology changes were observed with an inverted microscope. The expression of ZO-1was examined with western blot, and the distribution of ZO-1 was assessed by immunofluorescence. Actin cytoskeleton and focal adhesion (vinculin) were also assessed by immunofluorescence. Data were analyzed with commercial data analysis software and a p<0.05 was considered to be statistically significant.

**Results:**

There was no evident morphology change after 24 h culture in 60 mmHg pressure in iHTM and GTM_3_. However, in both iHTM and GTM_3_, elevated pressure attenuated the expression of ZO-1 at 12 h and 24 h, detected by western blot. Meanwhile, high pressure disrupted the organization of ZO-1, actin cytoskeleton, and vinculin, assessed by immunofluorescence. When comparing iHTM with GTM_3_, the distribution of ZO-1 and vinculin in GTM_3_ was not as regular as that in iHTM. After exposuring in elevated pressure, the changes in GTM_3_ were more obvious than that in iHTM.

**Conclusions:**

Sustained pressure elevation may directly damage trabecular meshwork cells by injuring ZO-1, cytoskeleton, and foal adhesions. And GTM_3_ was more susceptible to damage than iHTM. We suggest that elevated pressure seems to be not only the results of damaged TM, but also an important factor for the injury of TM cells, stop or reverse the process may help developing new target for the treatment of primary open angle glaucoma (POAG).

## Introduction

Glaucoma, one of the leading causes of irreversible blindness, afflicts millions of people worldwide [1,2]. Elevated intraocular pressure (IOP) and associated progressive optic neuropathy are the most important features of glaucoma [3]. Primary open angle glaucoma (POAG) is the most common form of glaucoma. Pathology changes of trabecular meshwork (TM) in POAG is largely responsible for the increased outflow resistance of aqueous humor, which will result in IOP elevation [4].

In the TM, arrays of collagen beams are covered by endothelium-like cells, that is TM cells. The TM cells in glaucoma patients have histopathology abnormalities compared with normal people [4,5]. At the same time, TM cells were found to be fewer in glaucoma patients compared with that in normal people [6]. Although several potential mechanisms have been suggested, including oxidative stress, mitochondrial dysfunction, cytotoxicity, and cell apoptosis [7-10], the exact mechanism remains unknown.

Cell–cell adhesion molecules are integral membrane proteins that are associated with peripheral membrane proteins to regulate and integrate cell–cell adhesion-related phenomena. In multicellular organisms, cell–cell adhesion is critical for development and morphogenesis. It is generally believed that tight junctions (TJs) can dynamically alter their structural and functional properties under different conditions and are subject to modulation by a variety of cellular and metabolic regulators [11-13].

A key component of junctional complexes that regulates TJs formation is zonula occludens-1 (ZO-1), which is a 210–225 kDa protein of the membrane-associated guanylate kinase homologs gene family [14,15] and is shown to be indispensible for TJ formation. ZO-1 knockout (Tjp1^−/−^) mice showed delayed growth and development, and ZO-1 may be functionally important for cell remodeling and tissue organization [16]. The actin cytoskeleton is a highly dynamic network, whose functions is to mediate a variety of essential biologic functions in all eukaryotic cells, including intra- and extra-cellular movement and structural support. Focal adhesion and adherens junctions are membrane-associated complexes that serve as nucleation sites for actin filaments and as cross-linkers between the cell exterior, plasma membrane and actin cytoskeleton [17]. Therefore, ZO-1, cytoskeleton, and focal adhesions may play an important role for the tissue and the microenvironment maintenance.

In this study, we focused on the effect of elevated pressure on the expression and distribution of ZO-1, cytoskeleton and focal adhesion for the first time in TM cells.

## Methods

### Cell culture

The cell line of immortal human trabecular meshwork cells (iHTM) was kindly provided by Dr. Vincent Raymond (Laboratory of Ocular Genetics and Genomics, Quebec, CA) and glaucomatous human trabecular meshwork cells (GTM_3_) were obtained as a gift from Prof. Yuhao Peng (glaucoma research, Alcon laboratory, Fort Worth, TX). Cells were cultured in Dulbecco's Modified Eagle Medium: Nutrient Mixture F-12 (DMEM/F12; Gibco, Invitrogen, Carlsbad,CA) containing 15% fetal bovine serum (FBS; Gibco, Invitrogen) at 37 °C and 5% CO_2_. Cells of identical passage were incubated in serum free medium for 24 h before they were divided into two groups: control cells are incubated in the conventional culture incubator at atmospheric pressure for 6, 12, and 24 h; the elevated pressure group cells are incubated in the pressure system simultaneously.

### Pressure system

An incubator, which is similar with other studies [18,19], was designed to expose the cells to elevated hydrostatic pressure. The pressure chamber was connected via a low-pressure two-stage regulator to a certified source of 5% CO_2_/95% air. This arrangement provided constant hydrostatic pressure within 5 mmHg. The pressure chamber were maintained at 37 °C by placing them inside an electronically controlled conventional incubator. Pressure was monitored using a diaphragm driven dial pressure gauge plumbed into the inlet circuit adjacent to the pressure chamber inlet. This pressure gauge was readable through a window present in the door of the pressure system. To examine the cellular responses in different time point, elevated hydrostatic pressure (60 mmHg) was maintained for different time course. Cells of different groups were observed and photographed under an inverted microscope (Axiovert 40C; Carl Zeiss, Germany) equipped with a digital camera (PowerShot G9; Canon, Tokyo, Japan).

### Immunofluorescence analysis

iHTMs and GTM_3_ were incubated in chamber slides at a density of 2,000 cells/chamber and cultured in conventional incubator and pressure system, respectively, for 24 h. Then they were taken and fixed with 4% paraformaldehyde (PFA) for 15 min at room temperature, and then permeabilized in 0.1 M PBS containing 0.2% triton X-100 for 5min. After blocking the cells with 5% bovine serum albumin (BSA), they were incubated overnight at 4 °C with mouse antibody against ZO-1 (1:50; Invitrogen, Carlsbad, CA) diluted in 1% BSA in PBS. After washing in PBS, the slides were incubated for 1 h at room temperature with FITC goat anti-mouse IgG secondary antibody (1:50; Multiscience, Hangzhou, China) in 1% BSA.The cells were counterstained with hoechst 33342 (1:1,000; Molecular Probes, Invitrogen, Carlsbad, CA). Cells were then washed and mounted in anti-fade fluorescence mounting medium (Applygen Technologies, Beijing, China). Images was visualized using a Zeiss 100M confocal microscope (Carl Zeiss Jena GmbH, Jena, Germany).

### Western blot analysis

Cells at a density of 2×10^5^ cells/ml were plated onto 100 mm plates. After culturing in serum free medium for 24 h, they were incubated in a conventional culture incubator or in the pressure system, respectively, for 12 and 24 h. Then, they were rinsed and lysed in mammalian cell lysis reagent (Sigma-Aldrich, St, Louis, MO) containing protease inhibitor cocktail (Sigma-Aldrich). Samples were loaded onto a 10% SDS–PAGE gel and electrophoresis at 80 V and 150 V for 2 h. Proteins were then transferred to a polyvinylidene difluoride (PVDF) membrane (Bio-Rad, Hercules, CA). After blocking with 1% BSA for 1 h, anti-ZO-1 monoclonal antibody (1:500; Invitrogen) and anti-β-actin antibody (1:1,500; Multiscience Biotech, Hangzhou, China) were applied and incubated at 4 °C overnight. Bands were visualized by incubation with a HRP-conjugated goat anti-mouse secondary antibody (1:10,000; Multiscience Biotech) and chemiluminescence substrates (ECL Plus; Perkin Elmer Inc., Covina, CA). Blots were developed and analyzed with Image J software. The protein levels were normalized with β-actin.

### Measurement of cytoskeleton organization and focal adhesions

Cytoskeleton organization and focal adhesion were visualized using the Actin Cytoskeleton and Focal Adhesion Staining Kit (FAK100; Millipore, Billerica, MA). Cells were treated the same as the Immunoﬂuorescence Analysis above except that cells were immunolabeled with anti-vinculin (1:200) at 4 °C overnight and incubated with FITC-anti mouse (1:200) and TRITC-conjugated phalloidin at room temperature for 1 h.

### Statistical analysis

Statistics were performed with Graphpad Prism 5.0 software (GraphPad Software Inc., San Diego, CA). All assays were performed in at least three separate experiments. Data were presented as mean±SD and evaluated by paired *t*-test. A p<0.05 was considered to be statistically significant.

## Results

### No obvious morphology changes of both iHTM and GTM_3_ under elevated pressure

**Figure 1:**
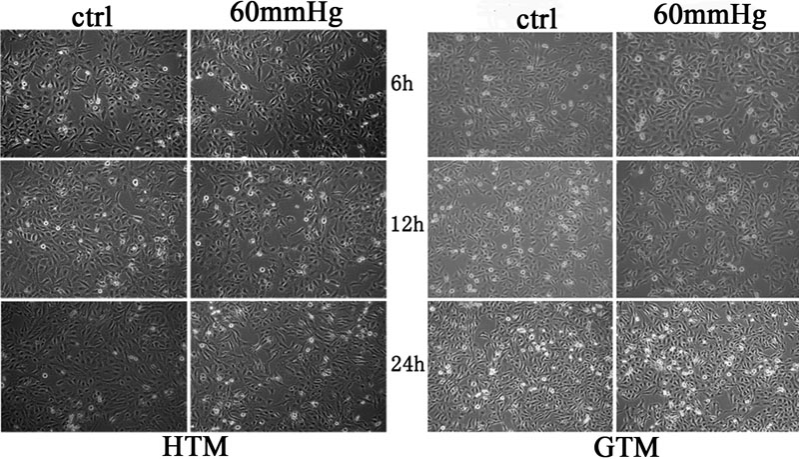
The morphology comparison of control cells and cells in 60 mmHg hydrostatic pressure. After treatment for 6 to 24 h, there was no significant morphology changes of iHTM and GTM_3_.

As is shown in Figure 1, we found that there was no evidence of morphological changes after exposure to 60 mmHg for 6, 12, and 24 h of both iHTM and GTM_3_.

### Expression of ZO-1 decreased in iHTM and GTM_3_ under elevated pressure

**Figure 2:**
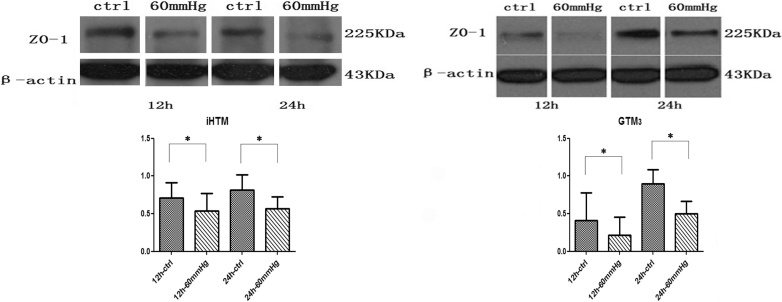
Western blot analysis of expression of ZO-1 in iHTM and GTM_3_ at 12 h and 24 h of culture in a conventional incubator and in a 60 mmHg pressure system. Elevated pressure caused the expression of ZO-1 decreased in both iHTM andGTM_3_ (*p<0.05).

Western blot analysis was performed to evaluate the expression of ZO-1 (Figure 2). In iHTM and GTM_3_, the expression of ZO-1 increased as time passed from 12 h to 24 h under normal pressure. While cells exposed to 60 mmHg hydrostatic pressure for 12 h and 24 h, the results showed statistically significant decreased expression of ZO-1 in both iHTM (12 h: 0.712 versus 0.540, p<0.05; 24 h: 0.813 versus 0.566, p<0.05) and GTM_3_ (12 h: 0.408 versus 0.214, p<0.05; 24 h: 0.901 versus 0.502, p<0.05) compared with that in control group.

The expression of ZO-1 in GTM_3_ was more distinctively decreased compared with that in iHTM (Figure 2, Table 1). In iHTM, the expression of ZO-1 attenuated to 74.33±13.34% and 69.32±3.04% in 12 h and 24 h, respectively, compared with the control group. However, in GTM_3_, it is 47.21±14.04% and 56.30±17.14%, respectively. All data were collected in at least three separate experiments.

**Table 1:** Relative ZO-1expression (normalized by β-actin) in iHTM and GTM_3_ at 12 h and 24 h culture in a conventional incubator (control) and 60 mmHg pressure system.

**Group**	**12 h-ctrl**	**12 h-60 mmHg**	**24 h-ctrl**	**24 h-60 mmHg**
iHTM (means±SD)	0.712±0.199	0.540±0.227	0.813±0.204	0.566±0.160
GTM_3_ (means±SD)	0.408±0.368	0.214±0.240	0.901±0.183	0.502±0.163

Every group of control and 60 mmHg is p<0.05.

### Elevated pressure caused disruption of normal ZO-1 distribution

**Figure 3:**
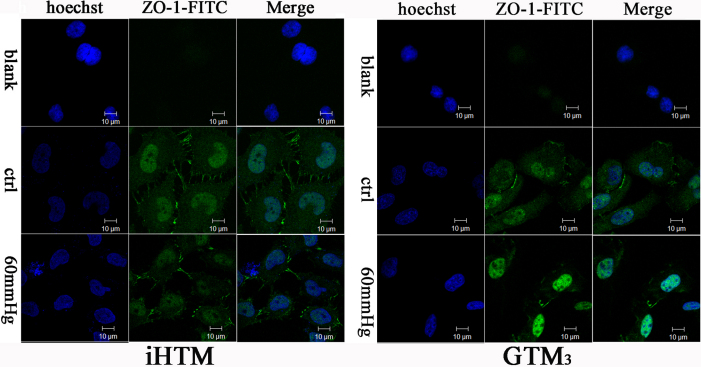
Effect of 60 mmHg hydrostatic pressure on ZO-1 in TM cells after 24 h. ZO-1 was immunolabeled with FITC and the nucleus was immunolabeled with hoechst, and observed under a confocal microscope, using identical parameters. The ZO-1 distribution in iHTM is clearly and regular, while that in GTM_3_ is irregular and tangled. Cells under 60 mmHg hydrostatic pressure showed significant decreased and irregular distribution of ZO-1 in both iHTM and GTM_3_ . The blank group showed that PBS was used instead of mouse antibody against ZO-1, other steps are the same. Every picture involved in the figure is the Z-depth slide that contain most green fluorescence (scale bar=10 µm).

Confocal laser scanning microscopy results (Figure 3) showed that in iHTM, the normal group had well distributed and clearly tight conjunction of ZO-1, while elevated pressure broke it into irregular distribution and the expression of ZO-1 decreased. Under normal pressure, GTM_3_ do not have the uniform and clearly ZO-1 distribution as it is in the iHTM, while the elevated pressure culture made it even worse.

### Elevated pressure caused collapse of cytoskeleton and disruption of focal adhesion in iHTM and GTM_3_

**Figure 4:**
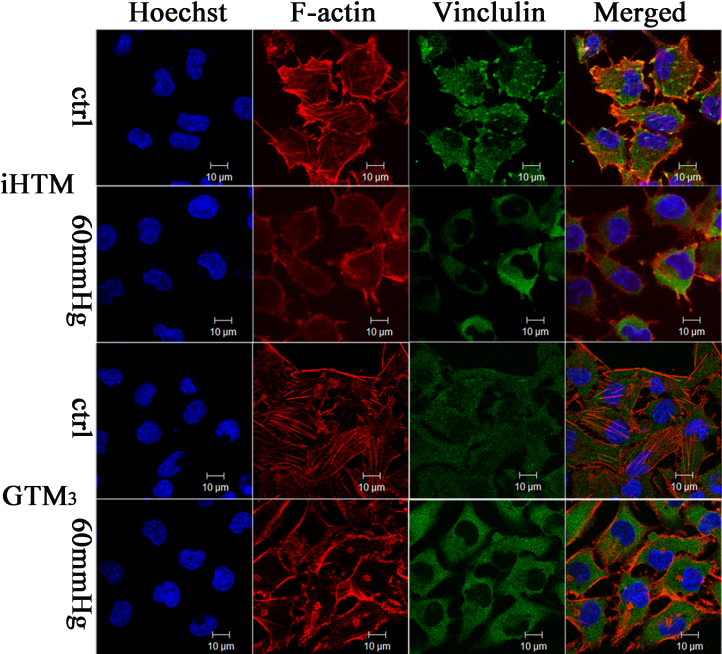
Elevated pressure (60 mmHg) induced collapse of cytoskeleton and focal adhesion in both iHTM and GTM_3_ after incubation for 24 h. Ctrl: cells cultured in a conventional incubator as the control group. 60 mmHg: cells cultured in the 60 mmHg elevated pressure system. The nucleus was immunolabeled with hoechst (blue fluorescence), filamentious actin (F-actin) was immunolabeled with TRITC (red fluorescence), and Vinculin was immunolabeled with FITC (green fluorescence). Slides were observed under the confocal microscope using identical parameters (scale bar=10 µm).

As illustrated by confocal laser scanning microscopy (Figure 4), both cell types were immunopositive to F-actin (red) and vinculin (green). The results showed that cells of both iHTM and GTM_3_ in control group (cultured in the traditional incubator) have polymerized actin in long, straight actin filament bundles. Cultured at 60 mmHg pressure for 24 h, the cytoskeleton collapsed and the actin filament bundles disappeared. In Figure 4, it is shown that the focal adhesion (vinculin) in control iHTM has numerous small dot-like focal complexes associated with actin filament bundles, but elevated pressure caused it to appear tangled, while GTM_3_ showed no clearly focal adhesion organization, either in control or in elevated pressure group.

## Discussion

Trabecular meshwork is known to be the most important pathway for the outflow of aqueous humor. Pathology changes of TM in glaucoma lead to increased outflow resistance of aqueous humor and IOP elevation [4,7-10]. Our results showed for the first time that elevated hydrostatic pressure could decrease the expression of ZO-1, disrupt the well organized ZO-1, cytoskeleton, and focal adhesions in TM cells. Oxidative stress, hypoxia and D-mannitol (high osmotic solution), nicotine and arecoline could also disrupt or influence the formation or arrangement of TJ protein (ZO-1) in many different kinds of cells [20-24]. Evidence indicates that oxidative stress may trigger degeneration in human TM, which affects the cytoskeleton and the adhesive properties in TM cells, subsequently leading to an increased IOP [25]. Instead, we found that elevated pressure in turn can also affect the cytoskeleton and the adhesive properties in TM cells.

Tight conjunction can control ion selectivity and permeability of the paracellular pathway between adhering cells [26]. Lack of ZO-1 had been reported to lead to changes of morphology and gene expression in corneal epithelial cells, which could destroy the corneal epithelial cells’ barrier function [22,27]. The environment of cells influence their adhesion, migration, differentiation, organ development, and death [28]. Elevated pressure induced disruption of tight conjunction may cause changes of the microenvironment and damage to the TM cells, which will also destroy the normal outflow of aqueous humor.

ZO-1 is a peripheral membrane adaptor protein of membrane-associated guanylate kinase homologs family with binding domains to adherens and tight junction proteins, in addition to the actin cytoskeleton [26,29]. In our study, we found that elevated pressure disrupted the F-actin cytoskeleton and focal adhesion, which is consistent with the decreased expression and the disrupted distribution of ZO-1. The serial effect of elevated pressure may also influence the cells adhesion on collagen beams, which may partially account for the reason why glaucoma patients have fewer TM cells compared with normal people.

Vinculin, as a focal adhesion protein, couples the extracellular matrix (ECM) through integrins to the actomyosin cytoskeleton. Recently, it has become a key protein regulating the transmission of contractile forces [30]. The amount of traction that a cell can develop is limited by its surrounding environment [31]. The ability to generate and transmit forces depends on the strength of the connection between the integrin adhesion receptors and the actomyosin cytoskeleton mediated by focal adhesion proteins. It is confirmed that contractile force generation is reduced when vinculin is absent, or enhanced when vinculin is present [28]. Here, we found that elevated pressure decreased vinculin, which may influence the contractivity of TM cells, and lead to high outflow resistance of aqueous humor. Maybe the effect of high pressure on the damage to ZO-1, tight junctions, vinculin, and cytoskeleton are a cascade reaction.

Moreover, a recent study suggested that tight junctions also participated in signal transduction mechanisms that regulate epithelial cell proliferation, gene expression, differentiation, and morphogenesis [29]. The function of focal adhesions are structural, linking the ECM on the outside to the actin cytoskeleton on the inside. They are also sites of signal transduction, initiating of signaling pathways in response to adhesion. Decreased expression of ZO-1 and disrupted focal adhesion induced by elevated pressure could also lead to some other signal changes and intracellular signaling cascades in TM cells, but the exact mechanisms require further exploration.

However, there was no evidence of TM cells death upon elevated pressure in this study. In the study, we choose to use no serum in the culture of TM cells because it will avoid the influence of serum and lead to more accurate results. While at the same time, long time incubation without serum may cause some nutrition deprivation effect, which will influence and be confused with the effect of elevated pressure, that’s why we cultured for only 24 h.Therefore,we speculate that the decreased expression of ZO-1 and cytoskeleton might be the primary change under elevated pressure. The process might be involved in maintenance of normal IOP at a stable level, by serving as a negative feedback, while after long time exposure in the pathological elevated pressure, apoptosis or cell death might happen.

In a previous study by our group [32], we found that dexamethasone increased the protein levels of ZO-1 in TM cells, which may have some relation with the increased outflow resistance of aqueous humor and high IOP in glucocorticoid-induced glaucoma. Here, on the other hand, we concluded that high pressure disrupted and decreased the expression level of ZO-1,which seemed to be conflict with previous study, but we speculate the disruption of ZO-1 by high pressure maybe the primary and initial reaction of TM cells and the further disruption of TM accounts for the elevation of IOP in POAG. All the results lead to the notion that ZO-1 may play an important role for the physical circulation of aqueous humor and the maintenance of normal IOP in different styles of glaucoma.

In summary, this is the first time to show that elevated hydrostatic pressure injured TM cells by decreasing ZO-1 expression and destroying cytoskeleton and vinculin. Moreover, GTM_3_ seemed to be more vulnerable than iHTM when exposed to the same level of elevated pressure. Our data suggests that elevated pressure seems to be not only the results of damaged TM, but also an important factor for the injury of TM cells. We suggest there might be a deteriorate circulation in TM cell degeneration and IOP elevation in glaucoma patients, stop or reverse the process may have the potential role to help developing new target for treatment of POAG. Further investigation to assess molecular responses of TM cells to elevated hydrostatic pressure, both in vivo and in vitro, may contribute to explore the pathogenesis and progression of TM degeneration in glaucoma.
